# Supplementary figures and images for: Extracellular Vesicles Are More Potent Than Adipose Mesenchymal Stromal Cells to Exert an Anti-Fibrotic Effect in an In Vitro Model of Systemic Sclerosis
Source: Int J Mol Sci. 2021 Jun 25;22(13):6837. doi: 10.3390/ijms22136837 (PMC8269376; doi:10.3390/ijms22136837)

## Supplementary Material

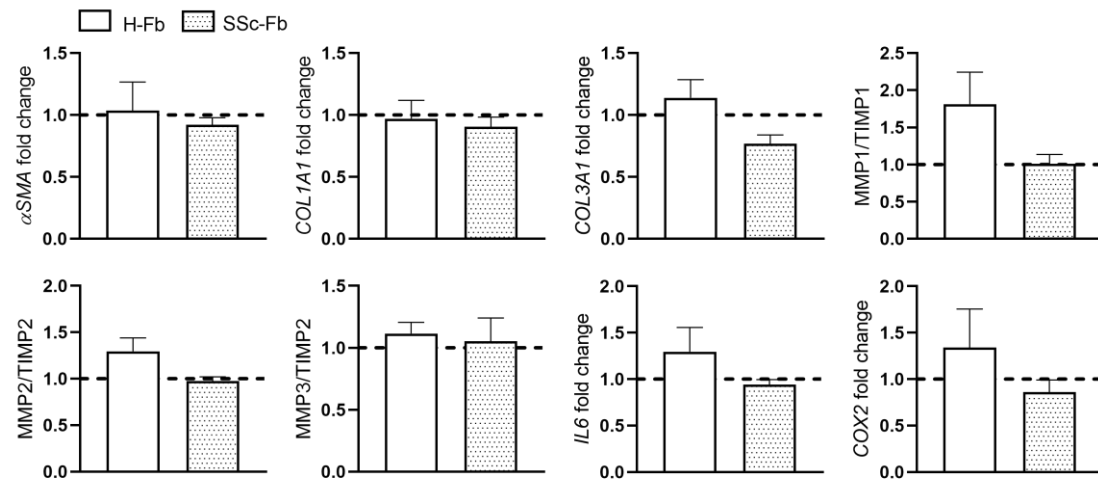

**Figure S1.** H-Fb or SSc-Fb that were not stimulated with TGFβ1.

Supplement: Supplementary file 1 [file ijms-22-06837-s001.zip › ijms-1272551-supplementary.pdf]
